# Barriers to Changing Sedation Practice for Patients Undergoing Mechanical Ventilation on the Intensive Care Unit: A Qualitative Interview Study of Clinical Staff

**DOI:** 10.1111/nicc.70348

**Published:** 2026-01-14

**Authors:** Nicholas D. Richards, Hilary L. Bekker, Simon J. Howell

**Affiliations:** ^1^ Adult Critical Care, Leeds Teaching Hospitals NHS Trust Leeds UK; ^2^ Leeds Institute of Medical Research University of Leeds Leeds UK; ^3^ Leeds Institute of Health Sciences University of Leeds Leeds UK

**Keywords:** clinical decision‐making, ICU sedation, intensive care, sedation practice, thematic analysis

## Abstract

**Background:**

Medical sedation is a requirement for mechanical ventilation for most patients admitted to the intensive care unit (ICU). Sedation can help minimise patient discomfort, pain and distress, but can lead to hypotension, bradycardia, prolonged ventilation and delirium. Improving sedation practice is key to improving sedation‐related patient outcomes.

**Aim:**

This study aims to explore ICU staff experience with sedation practices and identify potential areas for improvement and innovation.

**Study Design:**

Semi‐structured interview study exploring views, experiences and clinical decision‐making of ICU medical and nursing staff from two NHS adult ICUs in Yorkshire, England. Interviews were recorded to enable anonymous transcript production. Transcripts were coded using reflexive thematic analysis guided by Braun and Clarke's six‐stage method.

**Findings:**

We interviewed 18 members of ICU medical and nursing staff and using thematic analysis four interrelated themes were identified. First, staff perception of sedation, including understanding and reasoning around sedation goals. Second, the impact that ICU culture has on sedation practices. Third, sedation education and training for clinical staff. Finally, motivation to change, aspects impacting the delivery of sedation practices on ICU and key considerations for innovating change.

**Conclusions:**

Sedation practices in ICU are shaped by complex interactions between clinical framing, cultural norms, education and training and organisational pressures. Optimising sedation and implementing innovation require prioritisation of sedation as an active, goal‐directed treatment, supported by structured education and leadership engagement.

**Relevance to Clinical Practice:**

This analysis offers novel insights into barriers and facilitators to innovating sedation practices for mechanically ventilated patients, highlighting under‐prioritisation in clinical practice and training, cultural barriers and external influencers like staff retention and workload.

## Impact Statements

- What is known about the topic
  ○ Current international guidance suggests employing a multimodal, multidisciplinary approach to tailor analgesia and sedation to individual patient needs.
  ○ The adverse effects associated with sedation can significantly impact both patients and the NHS.
  ○ There is a lack of work investigating barriers to implementing sedation interventions, the majority of which have not considered the multidisciplinary approach to sedation, have not investigated barriers to new sedative agents and were conducted outside of the UK context.
- What this paper adds
  ○ This analysis illustrates how sedation is under‐prioritised in daily practice as well as in education and training. Staff perception, combined with ICU culture, not only limits individualisation of care and impedes innovation but also functions as a safety mechanism.
  ○ Improving sedation practice requires a complex intervention that addresses cultural barriers and the under‐prioritisation of sedation in current practice, while increasing sedation education and training and considering external influencers like staff retention and workload.

## 1 Introduction

Providing and optimising sedation for critically ill patients on the intensive care unit (ICU) is a fundamental aspect of critical care and plays an essential role in allowing patients to tolerate invasive therapies such as mechanical ventilation. Sedation reduces discomfort, pain and distress but can cause adverse effects such as hypotension, bradycardia and prolonged ventilation [1, 2, 3, 4]. Additionally, sedatives, in particular benzodiazepines, significantly increase delirium in ICU, which causes distress to patients and relatives and puts patients at risk of both short‐term and long‐term complications [5, 6, 7].

Current international guidance recommends a multimodal, multidisciplinary approach to tailor analgesia and sedation to individual patient needs, and, where possible, to minimise patient exposure to sedation while ensuring patients are pain free, comfortable and easily roused [2, 3, 8].

The adverse effects associated with sedation can significantly impact both patients and health services through increased mortality, increased length of stay and impeding patients' recovery and rehabilitation [9].

### 1.1 Justification for Study

Although research on standardising sedation practices exists, there is limited exploration of the barriers to innovating sedation practice [8, 9]. Historically, the contextual factors influencing the implementation and sustainability of sedation interventions have received less attention. Previous research has focussed on quantitative measures of sedation practices, such as the successful implementation of protocols and guidelines, rather than the reasons behind their success or failure [10, 11]. Only a small proportion of previous work employed qualitative design, leading to Varga and colleagues describing a ‘dearth of qualitative evidence’ in their 2022 systematic review of ICU staff perceptions of sedation practices [11].

Most existing studies have explored the barriers to implementing daily sedation interruptions or sedation protocols [12, 13, 14] and investigated staff perceptions of pain management [15, 16, 17]. Of the available studies, few considered the multidisciplinary approach to sedation, and no studies investigated barriers specific to implementing a new sedative agent or regime.

This study aims to investigate ICU staff views and experiences towards sedation practices. It explores the potential barriers to innovation, and how a sedation intervention may fit within the context of current ICU sedation practices. It forms part of a programme of work to develop and evaluate a complex intervention to improve sedation practices for mechanical ventilation in ICU settings [18, 19].

## 2 Study Design

### 2.1 Aims and Objectives

#### 2.1.1 Aims

The aim of this was to explore health professionals' views and experiences of sedation practices for mechanically ventilated patients in the intensive care unit.

#### 2.1.2 Objectives

1. Describe health professionals' views and experiences of sedation practices in ICU.
2. Investigate factors influencing sedation decisions in ICU such as clinical guidelines, roles and training
3. Explore perceived barriers and facilitators to innovation in sedation practices.

### 2.2 Methodology

We employed qualitative methods conducted in intensive care units within two large urban hospitals in the United Kingdom (2023–2024). The consolidated criteria for reporting qualitative research (COREQ) guidelines were used to structure the paper content [20].

The study uses a pragmatic research paradigm, combining constructivist and interpretative description approaches to explore subjective experiences and generate actionable knowledge capable of informing clinical understanding. This was influenced by the understanding that the researcher, the experience and context are all influencing the interpretation [21, 22, 23, 24].

This approach enabled our analysis to focus on staff perceptions and insights that allowed theme development to orientate towards clinically meaningful understanding and explanations, with the aim of highlighting, for example, how staff make sedation practice decisions, manage uncertainty and balance competing priorities. Throughout our analysis, we maintained a constant connection between the theoretical themes and their relevance to the clinical context. Themes were developed not only for their conceptual coherence but also for their capacity to explain practice, highlight areas of inconsistency and identify opportunities for improvement in sedation practices.

### 2.3 Methods

We used semi‐structured interviews to elicit staff views and experiences on sedation and clinical reasoning in ICU settings. The study design, methods and results were discussed with oversight groups and underwent peer review for doctoral research at the University of Leeds.

The interview schedule (Supporting Information Table S1) was developed based on relevant literature and research guidance [3, 8, 9, 18, 25]. The schedule was piloted with ICU staff from each professional group to ensure adequate coverage and relevance to the context, including current sedation practices, decision aids, sedation training and education, interprofessional and multidisciplinary dynamics and patient focus.

The schedule guided the discussion, but participants were encouraged to raise issues and lead on relevant topics.

All interviews were conducted by NDR, either face to face (University of Leeds) or online (Microsoft Teams, Microsoft, Washington, USA). Interviews were recorded for the purpose of anonymous transcript production. No external transcription services were used. Data, codes and themes were managed within Nvivo 14 (Lumivero, Denver, USA).

### 2.4 Target Population and Sampling

*Purposive sampling* was used to recruit nursing and medical staff from two large general adult ICUs at National Health Service (NHS) teaching hospitals (Site A and Site B). A *sampling framework* was used to guide the sample size, and recruitment was continuously reviewed throughout the study period to ensure the data had sufficient *depth* and *richness* to address the research objectives [26].

#### 2.4.1 Inclusion Criteria

1. Clinical nursing or medical staff currently working in an Adult ICU in England;
2. Experience with mechanically ventilated patients requiring sedation;
3. Doctors—experience choosing and prescribing sedation, knowledge of effects (adverse and beneficial) and monitoring effects/dealing with side effects;
4. Nurses—experience nursing patients on sedation, making up and administering sedation and monitoring effects/dealing with side effects;
5. Aged over 18;
6. Willing to provide informed consent for participation and, where appropriate, voice recordings for transcript generation and direct quotations (anonymous).

#### 2.4.2 Exclusion Criteria

1. Self‐reported conflict of interests regarding sedative choice (e.g., regarding sedative choice and potential for financial gain through involvement with a pharmaceutical company or with regard to a professional relationship with the interviewer)

Braun and Clarke, along with other researchers, now recommend basing sample size for reflexive thematic analysis on data having sufficient *depth* and *richness* for comprehensive analysis, rather than the previous recommendation of *data saturation* [26, 27, 28]. The maximum sample size was, therefore, not fixed and the sample size was reviewed and assessed continuously throughout the study, with ongoing recruitment based on richness of data with reference to the sampling frame [26, 29].

### 2.5 Participation and Consent Process

Recruitment was coordinated through a clinical lead at each site. Study participation materials were emailed to staff via work mail lists with a minimum of two recruitment rounds at each site, separated by several months. Recipients were asked to contact the research team directly if considering participating. Informed consent was gained prior to undertaking any study activities.

There were no financial incentives for participants.

### 2.6 Data Analysis

NDR analysed the transcripts guided by the updated Braun and Clarke six‐phase method [27]. A predominantly inductive, bottom‐up approach was used to identify both semantic and latent codes, with deductive coding ensuring relevance to the research question. Each transcript underwent a minimum of two rounds of coding. Codes were constantly revised, combined and re‐coded based on discussion with the wider research team (NDR, HLB, SJH), relation to the research question and context across the entire data set.

### 2.7 Reflexivity Statement

Reflexivity is key to understanding and acknowledging the impact of the researcher viewpoint, clinical experiences and knowledge of the subject area. It helps others to determine the validity of findings [30, 31]. The research team comprises experts in critical care, medical decision‐making and anaesthesia; we acknowledge these experiences and expertise provide important contextual understanding but can also impact both data collection and analysis through assumed shared understanding and privileging certain clinical narratives.

To manage preconceptions during data collection, open‐ended questions and follow‐up prompts were used that encouraged participants to share their reasoning, practices and understanding.

During analysis, attention was given to how researchers' disciplinary perspectives may shape theme development. Initial coding prioritised participants' language and meaning, remaining close to the data before moving to interpretive levels of analysis.

Through sustained reflexive engagement across data collection and analysis, we sought to balance the benefits of clinical expertise with a critical awareness of its influence, strengthening the transparency, trustworthiness and interpretive rigour of the findings.

## 3 Ethics Statement

Ethical approval was granted by the School of Medicine Research Ethics Committee at the University of Leeds (MREC22‐004) on 13 June 2023 and the Health Research Authority (HRA) (IRAS 322789/23/HRA/1194) on 5 June 2023.

## 4 Findings

Following the completion of 18 interviews, the consensus among the research team was that there was sufficient depth and detail, from a broad range of perspectives, to allow for reflexive thematic analysis and recruitment was closed. The sample included representation from different health professional types, experience level and site (see Table 1).

**TABLE 1:** Job role and experience of recruited participants.

Characteristics	Number recruited	
	Site A	Site B
Junior nurse (band 5)	1	1
Senior nurse (band 6–7)	2	2
Junior medical trainee or equivalent NTG (≤ST4)	1	1
Senior medical trainee or equivalent NTG (ST5‐8)	3	3
ICU consultant	4	0
Total:	11	7

Abbreviations: NTG, non‐training grade; ST, Specialty Trainee.

Interview duration ranged from 47 min to 83 min (mean 64 min). Thirteen (72%) were carried out in person, and five interviews were conducted virtually using Microsoft Teams. ICU experience varied from newly qualified nurses with 6 months of clinical experience, through to ICU consultants and senior nurses with well over 10 years of experience with sedated patients (range: 0.5–33 years, median 9 years).

### 4.1 Overview of Main Themes

Through reflexive thematic analysis, we identified four interlinked themes that impact current sedation practice and represent potential barriers to innovation:

Theme 1: *Staff perception of sedation: aims, motivation and consequences* categorise utterances linked to staff perception of sedation, including how the clinical context can affect the reasoning around sedation, and the impact this has on the perceived goals of sedation. It includes codes around the effects staff commonly associate with sedation and sedation practices and how these influence clinical decision‐making. The two subthemes are ‘Perceived aims and goals*’* and *‘*Motivation to achieve goals*’*.

Theme 2: *The effect of ICU culture on sedation practices* categorises utterances linked to cultural practices embedded within ICUs and the influence these have on the delivery and innovation of sedation practices.

Theme 3: *Skills, education and training* categorise utterances of staff experience, confidence and skills around sedation and ICU practices, as well as the education and training needs of staff related to sedation.

Theme 4: *Motivation to change* categorises utterances linked to changing care practices and barriers to innovating sedation practice.

Where there are quotations relevant to a theme or concept, these are denoted in brackets in the text, for example: (Q1). The corresponding quotation can be found in Supporting Information Table S2.

### 4.2 Theme 1—Staff Perception of Sedation: Aims, Motivation and Consequences

This theme explores participant views and understanding of the purpose of sedation within the context of delivering care on ICU and how these shape clinical decision‐making, priority setting and practice.

While sedation is a fundamental aspect of ICU, the importance attributed to sedation and how it fits into a patient's overall care are dependent on how it is viewed within the care pathway. Participants distinguished between sedation for a primary therapeutic indication (i.e., when the sedation itself is a treatment) and sedation to facilitate other invasive treatments, most commonly mechanical ventilation. Importantly, this distinction fundamentally altered how participants perceived sedation and how it was integrated into clinical practice (Q1–2).

Sedation to facilitate other treatments was commonly viewed as secondary to other aspects of care and received less focused attention. Participants described sedation in this context as lacking clearly articulated goals or endpoints and being afforded lower priority compared with protocol‐driven conditions such as sepsis (Q3). This contributes to its perception as a ‘*background practice*’ and even a ‘*clinical afterthought*’ (Q4–6).

*Q3: I don't think we see sedation as like a specific clinical goal in the same way as we do like managing sepsis or cardiogenic shock…* [Site A Trainee 01]

The under‐prioritisation of sedation was reflected in participants' accounts of target setting. (Q7–8). When sedation targets are explicitly stated, achieving these goals is challenging (Q9). Participants explained that achieving and maintaining light planes of sedation comes with additional workload and difficulties. Fluctuating patient needs and the relative difficulty in recognising over‐sedation compared with under‐sedation further compounded these challenges (Q9–Q10).

Clinical reasoning about individual sedation focused on minimising awareness, maintaining comfort and reducing pain and agitation. These decisions were often influenced by patients' visible agitation or discomfort (Q11–13). Participants saw sedation as crucial for preventing the psychological impact on both patients and their relatives. Some noted that this approach might result in excessive sedation, inadvertently causing or worsening delirium and increasing psychological distress for ICU survivors (Q14–15).

Judgements about sedation needs of individual patients were considered important when making clinical decisions about sedation and were informed by factors such as the patient's clinical condition, ventilator synchronisation, illness stage and sedation risks (Q16–18). A significant factor impacting sedation practices was the concept of ‘*safety*’, which encompassed both safety at the individual patient level and safety across the ICU as a whole. Participants described making trade‐offs between optimal sedation for individual patients, maintaining safety across the whole unit and the practicalities of medical care (Q19–20).

Participants demonstrated good awareness of the short‐term negative effects of sedative agents, such as hypotension, and described exposing patients to the least amount of sedation possible to avoid adverse effects while still achieving the desired clinical effect (Q21). However, participants described balancing this with significant apprehensions around risks of clinical incidences in lightly sedated patients, such as accidental extubation, especially during low staffing or high clinical demands, which impact individual sedation decisions (Q22–23).

Some participants discussed the long‐term effects of sedation and ICU care on patients' recovery, particularly the impact of sedation on physical and psychological recovery, describing how an increase in awareness of these long‐term impacts influenced their views of the goals, benefits and burdens of ICU (Q24–26). Despite this, short‐term consequences of light sedation seemed to be weighted more heavily than the longer term impact in most decision‐making (Q26–29).

Q30: *…an ICU nurse likes nothing better than a still and sedated patient. It's lovely to look after, but it's not to the benefit of most of the patients* [Site B nurse 01]

Participants acknowledged that deeply sedated patients are easier to care for but recognised the conflict when it may not benefit the patient, for example, increasing sedation before routine care like washing or rolling patients (Q30–33). It also may be easier to increase the sedation when other aspects of work, such as preparing drugs or administrative work and documentation (described as metrics), compete for priority (Q34–36). This tension highlighted the increasing workload, particularly for nursing staff, as a key barrier to improving sedation, particularly when competing interests from other clinical tasks or goals are considered higher priority than sedation. Without increasing staffing, it may be difficult to improve practice (Q37).

### 4.3 Theme 2—The Effect of ICU Culture on Sedation Practices

This theme explores how historic and cultural practices impact participants' perceptions and experiences of current sedation practices, decision‐making and capacity to change practice.

Participants consistently described the strong influence ICU culture, established unit practices have on sedation. Participants acknowledged that individualised sedation practice is preferable to ‘one‐size‐fits‐all*’* approach but is dependent on staff understanding, experience levels and training and that in reality there are trade‐offs between the ‘best thing to do*’* and the ‘pragmatic thing to do*’* (Q38). Sedation agent selection illustrates this conflict. Rather than individualised agent selection, participants describe minimal variation away from the ‘unit norm’ of propofol and alfentanil, or occasionally other opioids, which were used ‘across the board*’*. Participants described the use of these agents as ‘just what's done*’*, attributable to care protocols and historic unit practices (Q39–41).

Q40: *It's so entrenched that, you know, propofol and alfentanil is just what's done…* [Site B Trainee 02]

Participants discussed ICU or team culture as being shaped by staff experiences, familiar practices and team conformity (Q42). This conformity was often viewed positively, with participants noting how cultural norms can enhance safety and reduce risk in high‐stress situations (Q43). Participants also described how reliance on cultural practices may hinder clinical decision‐making and prevent change (Q44–45). This may also reinforce the perception of sedation as a ‘*background’* practice (Q1–Q4) with participants acknowledging that sedation receives less conscious thought or critical evaluation compared to other aspects of patient care, describing sedation as ‘almost an afterthought*’* (Q5‐6).

The Covid‐19 pandemic may have impacted both ICU culture and contributed to reduced awareness of sedation depth and implications. Participants described experienced staff leaving ICU roles and accelerated staff turnover, resulting in the loss of mentorship for junior staff and a revived culture of over‐sedation (Q46–47).

Q47: *The difficulty is I've got about 30% new [nursing] staff. Lots of them inexperienced with medicines. My band sevens are junior, my band sixes are junior. And I think it's replicated in most, if not all of the ICUs…* […] *My concern is it's just at the same time as we're getting less experienced staff, we've also got less experience staff mentoring them, supporting them and teaching them*. [Site B Nurse 03]

Culture was widely seen as a significant barrier to change, necessitating ‘*real impetus*’ for change to overcome staff reluctance to abandon familiar practices (Q44–45; Q48). Participants also shared concerns that change may expose staff to additional risks (Q49–50). Conversely, participants identified the top‐down culture of ICU as beneficial for change, provided there was buy‐in from senior members of the team (Q51).

### 4.4 Theme 3—Skills, Education and Training

This theme explores how staff experience, education and training shaped participants' confidence in managing sedation and influenced clinical decision‐making.

The role of experience in developing expertise around sedation decisions was a dominant view. More experienced nurses managed sedation proactively and were comfortable with lightly sedated patients, whereas less experienced nurses lacked confidence, describing anxiety, fear and uncertainty in sedation decisions (Q52). These concerns were reinforced by educational messages that emphasised the risks of under‐sedation, contributing to a perception that lighter sedation increased personal accountability and exposure to criticism in the event of adverse events (Q50; Q53).

Q53: *I think there's definitely a lot of caution around sedation and that is a culture, and that is taught: “You've got to be really careful…”* [Site A Nurse 02]

Participants noted that experienced nurses leaving services resulted in a loss of support and mentorship for junior staff, impeding their ability to gain necessary knowledge and skills through mentorship and bedside teaching (Q47; Q54). Furthermore, participants expressed concerns that shortages of experienced staff and limited teaching may lead to incorrect or contradictory information being modelled, affecting new staff's confidence and spreading suboptimal practices (Q55–56).

Formal education on sedation was widely described as insufficient, inconsistent and lacking clinical depth and was described as insufficient for high‐quality care (Q57). Both doctors and nurses noted a lack of structured training. Formal teaching was inconsistent and brief, relying on experiential learning and informal ‘*word‐of‐mouth*’ teaching from senior colleagues (Q58–62).

Nursing participants described sedation training as superficial, with limited opportunity to apply knowledge to the clinical setting (Q63). This reinforced reliance on experiential learning and informal knowledge transfer, rather than evidence‐based understanding. Medical participants similarly described their sedation training as informal and opportunistic, often dependent on self‐directed learning or ad hoc clinical teaching that was dependent on ‘*luck*’ (Q61; Q64).

Across both medical and nursing participants, there was a strong desire for more comprehensive sedation teaching; however, increasing clinical pressures were described as negatively impacting the delivery of all teaching, and additionally, sedation was then often not a prioritised topic for teaching (Q65–66). Familiarity with sedation evidence was consequently reported to be low, with participants prioritising research and learning in other areas of their practice (Q67–68).

Q57: *… I think that it [sedation training] is the necessary basics, but not sufficient for good care and for the avoidance of, or for the minimisation of problems associated with sedation*. [Site A Trainee 02]

Participants believed that enhancing sedation training could improve understanding of sedation's aims and goals, enabling them to titrate sedation more autonomously, allowing practices to become more patient focused (Q69). It may also reduce clinical practice inconsistencies, for example, discrepancies between documented Richmond Agitation Sedation Scale (RASS) and clinical findings (Q70).

Participants recognised that sedation practice is often influenced by staff expertise, linked to staff training, not just patient need (Q19; Q62). Inadequate education and limited clinical mentorship were identified as key barriers to optimising current sedation practice.

### 4.5 Theme 4—Motivation to Change

Theme 4 explored participants' views on innovating sedation practice, focusing on what motivates change and the conditions perceived as necessary for change to be feasible and sustainable.

Participants identified several issues with current sedation practices and expressed openness to innovations aimed at improving patient outcomes (Q71). Participants emphasised that recognition of the *need* for change was a critical first step and this was driven primarily by education and exposure to evidence demonstrating the rationale for change, particularly in relation to patient outcomes (Q71–Q73).

Q72: *I think the key for change is often people appreciating there's a problem in the first place. You may see that there's a problem, but what you need is buy in from the stakeholders that there's a problem*. [Site A Consultant 03]

Concerns about risk and safety were prominent when discussing introducing sedation interventions. Participants described apprehension about adopting new sedation strategies or agents without robust evidence and clear guidance, compared to familiar regimens with known safety profiles. As a result, participants emphasised the need for strong evidence, clear protocols and senior buy‐in to support change and mitigate individual risk. Once accepted, particularly by senior team members, participants felt the effects of culture could help other staff embrace necessary practice changes (Q74).

## 5 Discussion

Optimising and innovating sedation practices could significantly impact patient outcomes from ICU. Through reflexive thematic analysis, we identified four key themes that illustrate how clinical judgements, particularly around depth of sedation, involve complex interactions and shared understanding between the medical and nursing teams, balancing individual patient needs with the needs of the whole ICU and prioritising a myriad of clinical tasks expected of critical care staff.

The importance attributed to sedation, and how it fits into a patient's overall care, is dependent on how it is viewed by the clinical staff. Participants' accounts demonstrate that perceptions of sedation change with clinical context, describing a difference between sedation for a therapeutic indication compared to sedation where the purpose is to facilitate other treatments. When sedation is framed as facilitating other treatments, it is often no longer regarded as an ‘active treatment’ with explicit goals; instead, it becomes a background practice, competing for attention with other clinical priorities. This perception influences sedation decision‐making and, in combination with ingrained cultural practices, can lead to under‐prioritisation of sedation in clinical practice and a ‘*one‐size‐fits‐all*’ approach.

ICU culture emerged as a powerful influence on sedation practice, and while this approach can enhance safety, facilitate learning and prevent outlying practice, it may also reduce critical evaluation, prevent individualised care and present significant barriers to change.

These findings build on previous work that identified the perception of sedation or pain interventions as a priority in clinical practice as a vital component in optimising delivery [12, 15, 16]. This study adds new understanding of how cultural conformity simultaneously enhances safety while constraining innovation, something that has not been described in this context before. This dual role of culture helps explain why some sedation practices may persist despite contradictory evidence and explains why ‘top‐down’ approaches may be effective mechanisms for change in future service innovation approaches.

Participants consistently linked confidence in sedation management to experience and training. Less experienced nurses described anxiety and fear when caring for lightly sedated patients and participants across both professions expressing a need for more formal sedation training. Participants described current training is inconsistent and insufficient compared to other aspects of ICU practice. Improving training through classroom and bedside teaching would enhance understanding of optimal sedation practices, balancing the ‘*art*’ of sedation with the ‘*science*’ behind it.

Over‐reliance on experiential learning and peer‐teaching, particularly focusing on the risks of lighter sedation, may have led to the prioritisation of the short‐term ‘*seen’* costs of under‐sedation over the ‘*unseen’* long‐term effects of excessive sedation and the belief that deeper sedation mitigates short‐term clinical incidences. Coupled with an under‐appreciation of long‐term implications of deep sedation, and the recognition that deeply sedated patients are easier to care for, this aligns with prior studies that have identified beliefs, awareness and understanding of intervention aims and benefits as determinants of practice delivery and that deep sedation is easier [10, 12, 14, 17, 32, 33, 34, 35].

Prior work has demonstrated the impact of external factors such as the physical environment, workload and staffing levels has on optimal sedation practice delivery [10, 12, 13, 17, 32, 33, 34, 35]. This analysis adds to this, describing how these external factors not only negatively impact optimal sedation practice delivery but also limit the ability to deliver sufficient training.

These findings highlight how challenges in optimising and innovating sedation practice arise from the complex interaction of how staff perceive sedation, cultural practices, education and understanding around sedation interventions and organisational pressures that collectively shape everyday clinical decision‐making. Leadership endorsement and reframing clinical prioritisation of sedation relative to other clinical interventions and workload are key to implementing change.

### 5.1 Strengths and Limitations

This research study was designed to employ the well‐established Braun and Clarke six‐stage approach to thematic analysis, demonstrating dependability and confirmability. We sampled two independent sites to enhance credibility and transferability, and the research was conducted with rigour and reflexivity, increasing the credibility and confirmability of findings.

Despite multiple rounds of recruitment and efforts to recruit evenly across both sites using a sampling framework, no consultants were recruited from Site B. The reason for this and the impact it may have had on the findings are unclear. However, the views and experiences of both nursing staff and medical trainees were similar across both sites, which minimises the potential limitations imposed by the absence of consultants from Site B.

Although we have confidence in the validity of findings, further research should explore differences and similarities in practices and team needs across different contexts and should also consider other members of the multidisciplinary team that may have an influence on sedation practice, for example, advanced practitioners, physiotherapists and pharmacists.

### 5.2 Implications for Future Practice

This study has provided valuable new insights into sedation practices on ICU, highlighting key influencers of practice delivery and barriers to innovation.

The process of changing practice will involve asking staff to adopt an unfamiliar treatment regime. This will require innovators to address significant cultural barriers and educational gaps, which may be challenging and resource intensive. Additional external factors such as unit acuity, heavy clinical and non‐clinical workload and low staffing levels or high turnover of staff all impact the ability to deliver current sedation practices and therefore will also impede implementation of new practices.

The presented findings emphasise the importance of raising the clinical priority of sedation. Nurse leaders and advanced practitioners should actively promote sedation goals into daily nursing practice and endorse lighter sedation practices that align with current published guidance, while remaining conscious of the additional workload this may create for nursing staff.

Educators should move beyond experiential style learning for sedation. Structured, clinically relevant bedside and classroom‐based teaching may help reframe sedation as an active intervention requiring careful consideration and skilled titration. Making explicit the need for clinical reasoning about all medical interventions along a pathway of care, and the differential roles of the team, may support staff to think more proactively about opportunities to enhance patient‐centred practices.

Changing staff perception and prioritisation of sedation within ICU teams is key to both improving current sedation practice and innovating practice. Future sedation research should try to address the barriers described in this study during the planning phase of an intervention as this will be crucial for developing a sustainable intervention. Stakeholder buy‐in is key and could turn ICU culture into an advantage, utilising top‐down, senior‐led implementation.

## 6 Conclusion

The provision of sedation is a fundamental aspect of critical care and the delivery of sedation practices significantly impacts patient outcomes and experiences and accounts for a significant proportion of clinical staff workload [2, 3, 4, 5]. Despite recommendations for light sedation, achieving it consistently is challenging, even in trials [36, 37]. Additionally, the incidence of adverse effects linked to sedation such as delirium and post‐ICU weakness and psychological ramifications remains high [38, 39].

This rigorous analysis explored health professionals' views and experiences of sedation for mechanically ventilated patients on ICU. We have highlighted the complex interactions between clinical context, staff perceptions, cultural factors, external factors limiting optimal sedation practice delivery and the trade‐offs staff make between the needs of an individual patient versus the needs of the ICU as a whole.

Efforts to improve or innovate sedation practices must extend to addressing how sedation is conceptualised, taught and operationalised in ICU. Improving clinical staff understanding of *what* should be done, *why* it should be done and *how* it can be achieved is essential to improving outcomes for patients. As a collective, we must also address external influencers that restrict the delivery of optimal sedation practice, such as staff retention, skill mix and workload.

## Funding

This work was supported by NDR's time is part funded by the Leeds Doctoral Scholarship (University of Leeds).

## Ethics Statement

Ethical approval was granted by the School of Medicine Research Ethics Committee at the University of Leeds (MREC22‐004) and the Health Research Authority (HRA) (IRAS 322789, 23/HRA/1194).

## Consent

Informed consent was obtained prior to any research activities commencing.

## Conflicts of Interest

The authors declare no conflicts of interest.

## Supporting information


**Table S1:** Interview schedule and topic guide.
**Table S2:** Example participant quotes.

## Data Availability

The data that support the findings of this study are available from the corresponding author upon reasonable request.
