# Supplementary material for: Barriers to Changing Sedation Practice for Patients Undergoing Mechanical Ventilation on the Intensive Care Unit: A Qualitative Interview Study of Clinical Staff
Source: Nurs Crit Care. 2026 Jan 14;31(1):e70348. doi: 10.1111/nicc.70348 (PMC12805316; doi:10.1111/nicc.70348)
Supplement: Supplementary file 1 — Table S1: Interview schedule and topic guide. Table S2: Example participant quotes. [file NICC-31-0-s001.docx]

| **Topics** | **Examples of questions and probes** |
| --- | --- |
| ICU clinical experience | - What is your job role? - How long have you worked within ICU? - How would you describe your level of experience, particularly with regards to sedated patients? |
| Current sedation practice   - - Guidelines and protocols   - Individual (and individualised) practice   - Goals / aims   - Evidence and beliefs affecting decision making | - What is your understanding of sedation aims and goals? - Are there sedation guideline or protocols that are employed here / that you follow? - What is “first line” or most commonly used? Why? - What sedatives do you commonly prescribe / administer? - Do you ever prescribe / administer sedatives outside of protocols / guidelines? When/why? *(e.g. specific circumstances / patients)* - What factors do you consider when choosing a sedative? *(e.g. patient, safety, risks, costs, cultural)* - Are you aware of any evidence with regards to ICU sedatives? Does it alter your decision-making? - Describe the process of looking after a sedated patient as an ICU nurse / doctor? What decisions are you making and what goes through your mind? - Sedation targets - who sets targets? What is the goal? Are they met? Why / why not? - Issues around current sedation practice? - What would a good sedation regime look like? What would be a good outcome? |
| Education and training | - What teaching or training have you received around sedation and sedatives? - What kind of education is needed around sedation? |
| Innovation | - - Anticipated barriers to change? *(e.g. attitude, organisational, cost, patient-based)*   - Practicality barriers   - Organisations barriers |

# Supplementary Materials

Table S1 – Interview schedule and topic guide

**Table S2 – Example participant quotes**

| Quotation Number | Quotation |
| --- | --- |
|  | *“I think in the patients who are sedated for ventilation, I don't really think we think about it as a treatment per se. It's just something we do to facilitate ventilation… […] I don't think we really regard it as a treatment, just more of a kind of requirement.*” [Site B trainee 04] |
|  | *“I see it [sedation] as important in terms of facilitation of care. I don't strictly see it as a treatment, I don't see it as an active treatment…”* [Site A Trainee 05*]* |
|  | *“I don't think we see sedation as like a specific clinical goal in the same way as we do like managing sepsis or cardiogenic shock…”* [Site A Trainee 01] |
|  | *“It [sedation] just seems like a background ICU thing…”* [Site B Trainee 01] |
|  | *“… yeah, to the point where we probably don't pay as much attention [to sedation] as we would other things like the ventilator or the vasopressors. […] I think it's just one of those things that it’s running in the background and we kind of forget about it, because there’s lots of other things to focus on.”* [Site B Trainee 03] |
|  | *“I think it's something that's almost an afterthought on a lot of medical decision making and ward round targets. […] we often don't think about sedation or RASS targets, and if we do, it's probably just kind of at the end, as a bit of an afterthought. Not at the forefront of our minds like blood pressure, oxygen levels, things like that. […] sometimes we get preoccupied with the others [clinical aspects] and never get around to it [sedation].”* [Site A Trainee 03] |
|  | *“… there doesn't seem to be any target RASS setting.”* [Site A Nurse 02] |
|  | *“I don't prescribe a sedation level; I don't think it's done by almost anyone.”* [Site B Trainee 04] |
|  | *“If it was really easy to get someone to a prescribed depth of sedation and keep them there, and that they would be safe regardless of what's happening, I think it would happen all the time. But I think because… because it's so fluctuant as to what's happening to that patient the sedation needs to react to that.”* [Site A Consultant 02] |
|  | *“My biggest concerns with the sedation and over sedating people, which is probably the thing that I'm worried about most… it's really easy to see if you're under sedating somebody. If you're over sedating, it's quite hard to see that because the patient looks calm and they're not causing anyone any real problems. And it probably goes on for a long time. I worry about the long term sequalae of their sedation.”* [Site A Cons 03] |
|  | *“… if you thought they were showing some signs of pain or discomfort, then you could maybe give them a bolus of that [sedation], or increase that [sedation] slightly.”* [Site A Nurse 02] |
|  | *“I think the end point is sometimes how they [patients] look in the bed.”* [Site A Trainee 04] |
|  | *“… some nurses have told me that they don't want the patient looking untidy for the relatives…”* [Site B Trainee 03] |
|  | *“… something that I do commonly encounter is that perception of whether the patient suffering with awareness and that often leads to patients being a little bit more deeply sedated than maybe would be necessary for the clinical context…”* [Site A Trainee 01] |
|  | *“I suspect there's an association with excessive sedation, or sedation longer than necessary, with delirium and therefore probably ICU sort of psychosis or reported distressing symptoms recalled by the patient at follow up clinic.”* [Site A Consultant 04] |
|  | *“…the things that I would think of would be underlying pathology. So, what's brought them to intensive care in the first place, because that would change my management […]. Things affecting my decision would be, if it's things like a respiratory issue, they're more prone to coughing, not syncing with a ventilator, desaturating…”* [Site B Trainee 02] |
|  | *“It's [making decisions about sedation depth] factoring in what they're coming in with. I mean, do they need to be heavily sedated, you know… a brain bleed or something that you do need nice and still…”* [Site B Nurse 01] |
|  | *“I often think about it in what I'm trying to achieve for that patient in terms of facilitating their treatment. And I try to think about doing that and balancing it against the harms of either the treatment I'm giving them, or the what I perceive to be the de novo harms associated with sedation or intensive care.”* [Site A Trainee 02] |
|  | *“So I think depth… it depends upon seniority of both medical and nursing staff that are available. So absolutely it plays into that. It plays into the experience of the nurse at the bedside and experience of the sister that's in charge and the amount of nursing staff or allied healthcare professionals that are available to respond to a sudden change in conscious level.”* [Site A Trainee 04] |
|  | *“… it [sedation] also involves some more complex judgments about like safety across units and skill mixes of nurses and doctors on any one particular day. That means that sometimes that's harder to achieve, harder to do well than maybe it should be.”* [Site A Trainee 02] |
|  | *“I think even from a simple point of view, if you're a new nurse, you know that if there's less sedation, the blood pressure is going to be better…”* [Site A Nurse 02] |
|  | *“…If you're particularly busy on the unit and the staffing mix is either limited or poor, or not enough nursing staff… then, from a safety point of view to prevent the risk or reduce the risk of accidental extubations, you might deepen the sedation if you really didn't want that patient to [extubate]…”* [Site B Trainee 01] |
|  | *“If there's just you and another nurse in the bay and it's some big fella that's been quite heavily sedated on a lot of stuff, you're probably not wanting to do that [reduce sedation] by yourself. So, you've got to plan for your staffing and things as much as anything else.”* [Site B Nurse 01] |
|  | *“… when you look at what happens to people when they’re discharged from ICU. It is actually really upsetting and its soul destroying really. When you're trying really hard to save someone's life to think that they can spend the next year incredibly depressed and like anxiety and panic attacks…”* [Site A Nurse 03] |
|  | *“I think about post ICU syndrome with everyone I admit now. Every time I admit someone, I worry trade-off of burdens and benefits, and post-ICU syndrome probably taking a year to get over… and it's pretty hideous in terms of all the different domains...”* [Site B Trainee 04] |
|  | *“I don't think that’s [long-term effects] at the forefront of people's minds, but I think it's becoming more thought of with the sort of increase in understanding of post-intensive care syndrome.”* [Site B trainee 01] |
|  | *“I think a lot of people just think about the here and the now and saving that patient right now, and I do too most of the time...”* [Site B Trainee 01] |
|  | *“I’d say we've probably not very good at considering the long-term psychological ramifications of having people sedated”* [Site A Consultant 01] |
|  | *“…the vast majority of patients are deeper rather than lighter… because I think there is a false opinion that it's safer […] I think it's um, perceived to be safer. But realistically in the long term, if you look at all your patients as a cohort, then, I would say that it's probably less safe doing that.”* [Site B trainee 01] |
|  | *“…an ICU nurse likes nothing better than a still and sedated patient. It's lovely to look after, but it's not to the benefit of most of the patients”* [Site B nurse 01] |
|  | *“I think it's perceived that it's easier… well, it's not perceived, it definitely is easier to look after, for vast majority of times easier for a nurse or a doctor to look after a deeply sedated patient… It's very easy to turn up the sedation to keep someone asleep rather than in that sweet spot where you want them doing a bit, but not agitated.”* [Site B Trainee 02] |
|  | *“…if then people aren't coughing on sedation, if there's no movement or eye-opening when you’re performing cares. And in some ways, that makes patients much easier to look after, but obviously, physiologically, it's not great for them.”* [Site A Nurse 03] |
|  | *“I know a lot of people especially during cares want them as flat [sedated] as possible.”* [Site A Nurse 02] |
|  | *I think over time [for nurses] there has been a culture of getting all of your observations and writing done before you do anything else, because otherwise you are unable to do all those things and I completely understand that there is pressure to get those metrics done…”* [Site A Trainee 04] |
|  | *“Sometimes I think the nurses are probably doing a lot of other things. So then actually being very goal orientated with the sedation isn't a priority for them…”* [Site A Trainee 01] |
|  | *“I think one; it [reducing sedation] falls down the priority list. Two; many people aren't always as aware of it [need to reduce sedation] as they should be. And three; the friction between being able to do other stuff while patients are like settled, in inverted commas… and it's quite a powerful driver of keeping people more sedated than they probably should be.”* [Site A Trainee 02] |
|  | *“… the ICU nurses have got so much going on just to keep the patient alive and out of multi organ failure, or managing the multi organ failure, then all these like additional value-added, patient experience goals… Well, I think we're at the point where you where we can't just add in more to the ICU nurses’ workload… you need more people to deliver that, you know, exemplary level of care.”* [Site B Trainee 04] |
|  | *“… there's also questions about what's the best thing to do and what's the most pragmatic thing to do, because, um, the optimal sedative drug for that patient might not be the pragmatic one to give […] So you can look at the pharmacokinetic and pharmacodynamic profile of a drug and you can sort of select out one that is, you know, the like… what do you think's going to be the winner but if that can't be delivered by the end user, the nursing staff, it’s a moot point.”* [Site A Consultant 01] |
|  | *“…we've got our standard propofol and alf [alfentanil], and it just comes as care bundle, so when the patient comes in we don't really consider what sedation we are gonna give this patient, or which is the right treatment for them…”* [Site B Trainee 04] |
|  | *“It's so entrenched that, you know, propofol and alfentanil is just what's done…”* [Site B Trainee 02] |
|  | *“…we seem to routinely start similar agents across the board*…” [Site A Trainee 01] |
|  | *“… there is a lot of culture around sedation that builds up in an ICU. This to be fair as much driven by the medical staff as the nursing staff. […] ICUs will tend to stick with what they know…”* [Site A Consultant 02] |
|  | *“I think that [familiarity through culture] generally leads to less errors on the human factors side of things. […] especially out of hours when you panicked and stressed… using what you know, at doses that you know, that everyone's comfortable with in a stressful acute situation, there's probably a benefit to that as well.”* [Site A Trainee 03] |
|  | *“Yeah, I think it becomes cultural, doesn't it? So, I think unless there was… unless there was a real impetus to make a change, or to protocolise what we were doing, I think you’d find that people just kind of fall into what everyone else is doing... And you often see that when new consultants come in, they may do things slightly differently to start with, and that's really noticeable for a little while, and then everyone just converges on the mean. […] So, it's really interesting actually, how people just fall into the culture of the unit.”* [Site A Consultant 03] |
|  | *“I think mostly what will be tricky is culture change. […] My main concern about trying to get it [change] going would be about culture… ‘this is what we always do’…”* [Site B Trainee 01] |
|  | *“… it feels like the current moment is a combination of lack of institutional memory; lots of people who are very experienced have left, mixed with short staffing, and relatively junior nursing ratios. We are still sort of slow… sort of… slowly gaining a level of care, and a level of skill, and a level of attention to detail, for this sort of stuff, which has basically... has gone out the window because of the pandemic.”* [Site A Trainee 02] |
|  | *“The difficulty is I've got about 30% new [nursing] staff. Lots of them inexperienced with medicines. My band sevens are junior, my band sixes are junior. And I think it's replicated in most, if not all of the ICUs…* […] *My concern is it's just at the same time as we're getting less experienced staff, we've also got less experience staff mentoring them, supporting them and teaching them.”* [Site B Nurse 03] |
|  | *“[It is] difficult to change people's opinions and difficult to change people from what they're comfortable with and start something new.”* [Site A Trainee 04] |
|  | *“I think there's a bit of resistance to change, particularly amongst our nursing teams, because if something goes wrong, they tend to be the ones that get crucified for it.”* [Site A Consultant 01] |
|  | *“I actively say to my junior staff, remember once you take on the responsibility for titrating that medicine, you take the accountability.”* [Site B Nurse 03] |
|  | *“…I think if you can do that to senior team members, and get buy-in from them, then sort of the culture spreads.”* [Site B Trainee 01] |
|  | *“… initially it was really frightening… […] I'd even be scared to even touch… like… kink one of the lines accidentally or even titrate one of the values because you'll be afraid that you going to stop it and they’re going to wake up straight away. So, I think I think it's an anxiety that a lot of new people have…”* [Site A Nurse 02] |
|  | *“I think there's definitely a lot of caution around sedation and that is a culture, and that is taught: ‘You've got to be really careful…”* [Site A Nurse 02] |
|  | *“I'd say there's a lot fewer nurses that will have the confidence to increase or decrease sedation without running it past a doctor.”* [Site B Nurse 03] |
|  | *“…they [new starters] are at the bedside with a band five that doesn’t really know it themselves that goes ‘oh yeah I've told them everything I know’, but it's only the level that they know…”* [Site B Nurse 03] |
|  | *“… sometimes you get different opinions when you're on the floor so somebody's telling you one thing and then others telling the other thing, but in the classroom setting where somebody who is probably more knowledgeable who would have done their own in-depth learning will come and tell you ‘this is the protocol and this is why we do what we do and this is what we're looking for’. It's far better to take that learning to the floor so that when you're administering your care it will make more sense and you'll be able to use more of your judgment, more than um just hearing what person A is saying that contradicts to what person B is saying at the same time…”* [Site B Nurse 02] |
|  | *“… I think that it [sedation training] is the necessary basics, but not sufficient for good care and for the avoidance of, or for the minimisation of problems associated with sedation.”* [Site A Trainee 02] |
|  | *“… in terms of a kind of training point of view, there's not really that much around sedation from this point of view, you very much just… experience it…”* [Site A Nurse 02] |
|  | *“I remember talking to colleagues a lot to get a lot of my information. […] it was mostly based on experience and like other people's experience. […] that's just things that I’ve kind of picked up from other people really. […] it was very much like word-of-mouth practice, and being with somebody who had experience.”* [Site A Nurse 03] |
|  | *“I think it's something that at that junior level when you start on ICU, that you just pick up by watching rather than officially being taught…”* [Site A Trainee 03] |
|  | *“…I think teaching is a bit ad-hoc depending on where you work, as to how much you get, who delivers it, whether it's formal or informal.”* [Site B Trainee 01] |
|  | *“The nurse being really inexperienced or uncomfortable looking after someone who is lightly sedated would affect my decision about having a patient lightly sedated.”* [Site B Trainee 02] |
|  | *“I've done, a kind of online module study day on sedation, it's very much around the medications, but less so on, kind of, targets of sedation. […] So I think it was less so about how we manage and titrate sedation, but much more about these are the medications that we might use for sedation.”* [Site A nurse 02] |
|  | *“Maybe you'd be lucky to get teaching because it's the topic of the weekly teaching or something, but that that's obviously really variable.”* [Site A Trainee 03] |
|  | *“Yes I would say we need more in-depth sedation teaching, I do think there is a need, and the difficulty is as they need staff on the floor and the managers say clinical need comes before everything else.”* [Site B nurse 03] |
|  | *“I'd say it's [sedation] probably seen as less of a priority for teaching than how to work the ventilator, cardiovascular support, those things like that.”* [Site A Trainee 03] |
|  | *“I probably should know more… seeing as I can quote studies for the majority of stuff I do, it would be nice and I probably should… we probably should be able to quote a number of studies in terms of sedation, as it’s a big part of our day-to-day working…”* [Site A Trainee 04] |
|  | *“I'm not that familiar enough with the evidence base to be able to sort of make a very like academically informed decision on sedation.”* [Site A Trainee 01] |
|  | “This [improving sedation teaching] will give us better understanding, you know, to understand why we do this, you know, and then we can all be on the same page and I can sort get to that point where I'm confident enough I can suggest some things and be more autonomous, you know because I understand why we're doing what we're doing, and then what we're trying to achieve as well.” [Site B nurse 02] |
|  | *“When you examine patients that are on ventilators, the majority of them are a RASS level of minus four, but minus two will be written in the notes…”* [Site A Trainee 04] |
|  | *“… lots of experienced people would think that, and will have identified the same problems that I have with ICU sedation in UK intensive care practice and conclude that all of the things I've said are system, clinician, process problems, rather than just pharmacological problems.”* [Site A Trainee 02] |
|  | *“I think the key for change is often people appreciating there's a problem in the first place. You may see that there's a problem, but what you need is buy in from the stakeholders that there's a problem.”* [Site A Consultant 03] |
|  | *“I think if people understand why, you get by in, but I think if people feel like they are getting told to do stuff that they inherently think is dangerous, or makes their life harder, or deviates from the norm… and that's true of doctors, it's not just nurses… […] they just need to understand why you're doing it and I think then people are pretty receptive.”* [Site B Trainee 01] |
|  | *“…I think if you can do that to senior team members, and get buy-in from them, then sort of the culture spreads.”* [Site B Trainee 01] |
